# Optimizing Processing Parameters and Surface Quality of TC18 via Ultrasonic-Assisted Milling (UAM): An Experimental Study

**DOI:** 10.3390/mi14061111

**Published:** 2023-05-25

**Authors:** Guangxi Li, Weibo Xie, Hongtao Wang, Yongbo Chai, Shaolin Zhang, Liquan Yang

**Affiliations:** 1 Henan Province Engineering Research Center of Ultrasonic Technology Application, Pingdingshan University, Pingdingshan 467000, China; 2677@pdsu.edu.cn (G.L.); 15838190125@163.com (Y.C.); 2 School of Intelligent Manufacturing and Transportation, Chongqing Vocational Institute of Engineering, Chongqing 402260, China; 20180701045@cqu.edu.cn; 3 School of Mechanical and Power Engineering, Zhengzhou University, Zhengzhou 450001, China; wanghongtaozzu@163.com (H.W.); zhangshaolin@zzu.edu.cn (S.Z.)

**Keywords:** longitudinal ultrasonic-assisted end milling, TC18 titanium alloy, cutting force, residual stress, surface topography, milling temperature

## Abstract

This study conducted longitudinal ultrasonic-assisted milling (UAM) tests and optimized a combination of milling technological parameters to achieve high-quality machining of TC18 titanium alloy. The motion paths of the cutter under the coupled superposition states of longitudinal ultrasonic vibration and end milling were analyzed. Based on the orthogonal test, the cutting forces, cutting temperatures, residual stresses, and surface topographical patterns of TC18 specimens under different UAM conditions (cutting speeds, feeds per tooth, cutting depths, and ultrasonic vibration amplitudes) were examined. The differences between ordinary milling and UAM in terms of machining performance were compared. Using UAM, numerous characteristics (including variable cutting thickness in the cutting area, variable cutting front angles of the tool, and the lifting of the cuttings by the tool) were optimized, reducing the average cutting force in all directions, lowering the cutting temperature, increasing the surface residual compressive stress, and significantly improving the surface morphology. Finally, fish scale bionic microtextures with clear, uniform, and regular patterns were formed on the machined surface. High-frequency vibration can improve material removal convenience, thus reducing surface roughness. The introduction of longitudinal ultrasonic vibration to the end milling process can overcome the limitations of traditional processing. The optimal combination of UAM parameters for titanium alloy machining was determined through the end milling orthogonal test with compound ultrasonic vibration, which significantly improved the surface quality of TC18 workpieces. This study provides insightful reference data for subsequent machining process optimization.

## 1. Introduction

Titanium alloy (TC18) is a typical near-β phase Ti alloy with a nominal composition of Ti-5Al-5Mo-5 V-1Cr-1Fe. Due to an excellent combination of intrinsic qualities, including low thermal conductivity, high specific strength, and high corrosion resistance, Ti alloys show broad application prospects when used as structural components in the aerospace field and light bearings; however, Ti alloys are relatively difficult to process, which remains a bottleneck problem.

Gaining in-depth knowledge of Ti alloy product processing technology and technical parameters is of insightful guidance for engineering applications. Tej Pratap et al. established the cutting force model during the milling process with the Johson–Cook constitutive equation [1,2] and performed related simulations to analyze the stress distribution, temperature rise distribution, and cutting force during the machining process of Ti alloy (Ti6A14V); moreover, they combined tests to validate the established model and cutting force. Wang et al. milled Ti alloy (TA15) after the filling of cutting fluid and investigated the wear condition of the coated cutting tool, the surface topography of the workpiece, and the variation of cutting force during the manufacturing process [2]. According to their results, both the cutting force and the wear of the cutting tool increased slowly and then violently with milling time. The surface roughness of the processed workpiece was negatively correlated with tool wear [2,3].

Ultrasonic-assisted cutting exceeds ordinary cutting methods in Ti alloy machining. Based on the principle of multiple contacts and separations between the cutter tool and the workpiece, scholars established an analysis model of ultrasonic-assisted milling (UAM) [4,5,6]; by comparing the effects of various technological parameters on the cutting force and surface morphology during the Ti alloy machining process using two methods, it was concluded that ultrasonic vibration cutting technology was superior to the traditional technique. Scholars worldwide have employed ultrasonic-assisted cutting methods to examine the surface integrity of difficult-to-process materials [7,8,9,10,11], which can provide a new approach for surface morphology investigations, such as surface roughness, texture, and frictional wear. Verma et al. [12] conducted in-depth studies on the generation mechanism of temperature rise induced by axial ultrasonic vibration; based on the heat source model, they constructed a prediction model of temperature rise during ultrasonic milling considering the ultrasonic softening effect.

Several scholars performed orthogonal tests to determine the appropriate parameters to explore the effects of ultrasonic-assisted and ordinary milling treatments on the machining parameters of difficult-to-process materials [13,14,15]. Ying et al. conducted longitudinal–torsional ultrasonic-assisted milling (UAM) on Ti6A14V and compared the results on cutting force, residual stress, and cutting temperature with the values using traditional milling [16,17,18]; moreover, in combination with orthogonal test and signal–noise factor test results, they investigated the effects of processing parameters on residual stress and finally concluded that LTUV-M can effectively reduce cutting force and cutting temperature and enhance residual pressure stress on the surface.

Some scholars have combined analytical methods and finite element simulations to simulate cutting force and cutting temperature during the machining process of Ti alloys, which can reflect the influence tendency to a certain degree [19,20,21,22]. However, the simulation results can only partially approach the actual machining results. Most studies have focused on popular materials, such as Ti6A14V. Traditional Ti alloys’ machining methods and parameters no longer apply to TC18.

This study focused on TC18 materials and constructed a test platform under longitudinal ultrasonic vibration and end-milling compound conditions. With an orthogonal test, surface milling was performed, and the changes in the milling forces along various directions and the milling temperatures under ultrasonic vibration were measured. Moreover, the residual stresses and the surface morphology on the processed workpiece surfaces were measured. The preset test results provided specific application values for the ultrasonic-assisted processing of TC18.

## 2. Materials and Methods

### 2.1. Longitudinal Ultrasonic-Assisted Milling Characteristics

**Figure 1:**
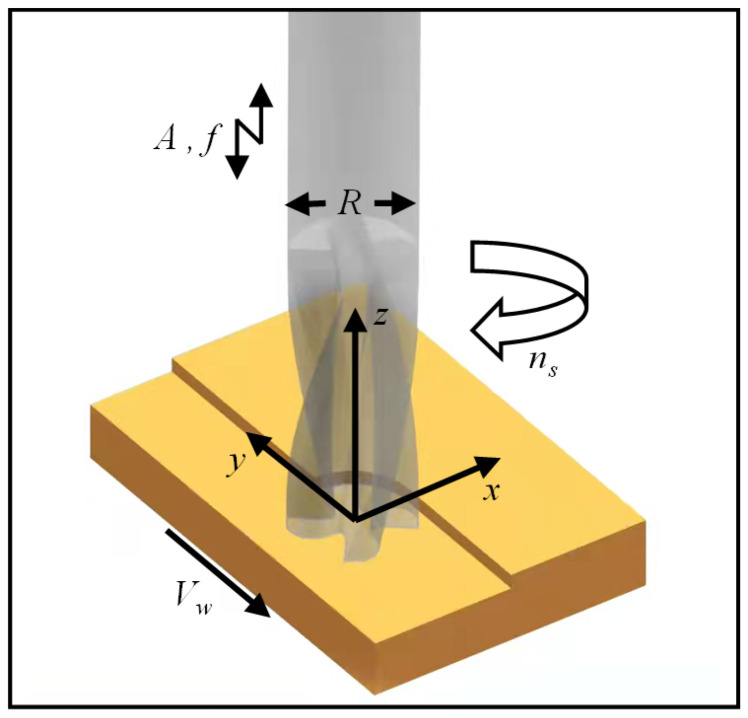
Illustration of the LUV-EM process.

Figure 1 illustrates the motion characteristics of the longitudinal ultrasonic milling tool, where R is the milling tool radius, ns is the tool rotation speed about the principal axis, *v_w_* is the tool feed rate along the *y*-axis, and A and f are the vibration amplitude and frequency of the longitudinal ultrasonic vibration, respectively.

During the longitudinal ultrasonic-assisted end milling process (LUV-EM), the milling tool can generate high-frequency periodic displacements along the axial direction. Combining conventional milling and longitudinal ultrasonic vibration can improve cutting force, residual stress, surface topography, and milling-induced temperature rise [23,24,25,26].

The motion path equation of the cutting edge in the LUV-EM system can be written as:

(1) $$\left\{ \begin{array}{l} x(t)=R\sin\frac{2\pi n_{s}t}{60}\\ y(t)=v_{w}t+R\cos\frac{2\pi n_{s}t}{60}\\ z(t)=A\sin(2\pi ft) \end{array}\right.$$

**Figure 2:**
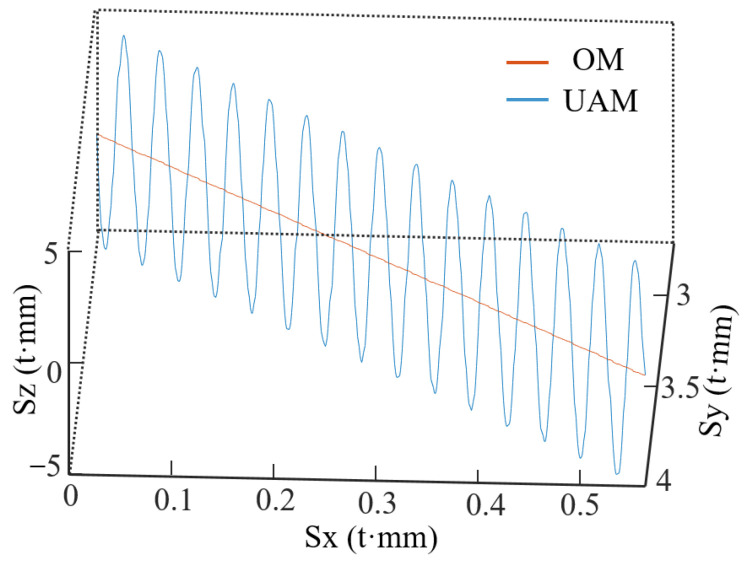
Comparison of motion paths using OM and UAM.

Figure 2 compares the motion paths of the milling tool using UAM and ordinary milling (OM). After adding longitudinal ultrasonic vibration, a sinusoidal waveform began to appear in the motion path of the cutting edge.

### 2.2. Test Device

**Figure 3:**
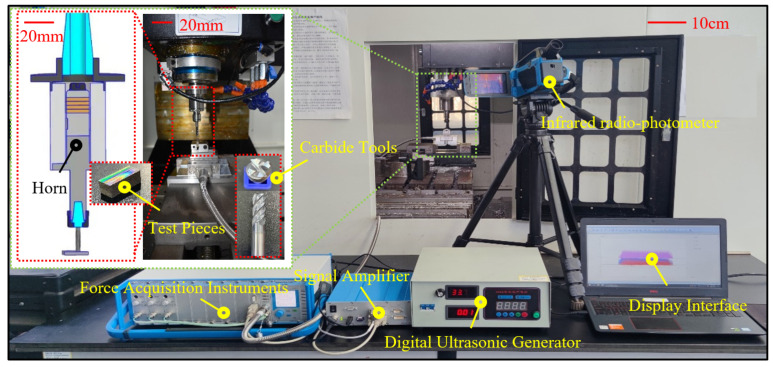
Illustration of the test device.

Ultrasonic milling of TC18 was performed in a CNC machining center (Henfux-HFM 700 L). As shown in Figure 3, the test device mainly consists of a wireless transmission system, ultrasonic tool holder, force measuring system, and temperature measuring system. During the operating process, the electric energy of the ultrasonic power supply is transmitted to the transducer via the wireless transmission system. The amplitude output from the transducer is then magnified by the amplitude transformer and transmitted to the cutter tool to mill materials. The force measuring system (Swiss Kistler Instruments Co., Ltd., Winterthur, Swiss) mainly comprises the milling force-acquisition system (Type 9119AA2), the signal amplifier (Type 5080A), and the display, while an IR imaging sensor (Type IRI-100C2, Xiamen Red Phase Electric Power Equipment Co., Ltd., Xiamen, China) was used to measure temperature.

### 2.3. Test Specimen and Tool Parameters

The TC18 test specimens were 15 mm × 6 mm × 8 mm in size. TC18 is an Al alloy consisting of α and β phases. Table 1 lists the chemical composition of the TC18 Ti alloy.

TC18 mechanical properties were as follows: tensile and yield strengths of 1220 and 1162 MPa, respectively; elongation of 17.4%; and cross-sectional reduction of 48.4%.

During the ultrasonic-assisted milling process, a cutter tool with favorable heat-conducting properties, swarf discharge performance, and a smooth coating was selected to prolong service life. Moreover, the coating needed to be composed of low-activity materials. This study used a 4-blade integral vertical milling cutter with hard-alloy columns (TM-4R-D8.0R1.0). The milling cutter was 8 mm in diameter. The blade and tool lengths were 20 and 60 mm, respectively. The cutter tool was coated with AlCrXN.

### 2.4. Test Design

To examine the effects of ultrasonic-assisted milling parameters, this study selected four parameters—the cutting speed *v_w_*, the feed *f_z_*, the cutting depth *a_p_*, and the ultrasonic vibration amplitude A, denoted as A, B, C, and D, respectively. We designed an orthogonal test to measure the cutting forces along three directions (*F_x_*, *F_y_*, and *F_z_*), the surface roughness after precision hard cutting (*R_a_*), the residual stress (*R*_s_), and the cutting temperature (*T*). We also calculated the results of variance and range. Table 2 lists the present test schemes and results.

After milling, the residual stress on the surface was measured with an X-ray diffractometer (PROTO-LXRD), while the surface micromorphology was observed under a COXEM EM-30 scanning electron microscope and a white light interferometer.

## 3. Results

This section may be divided by subheadings. It should provide a concise and precise description of the experimental results and their interpretation, as well as the experimental conclusions that can be drawn.

### 3.1. Cutting Force

**Figure 4:**
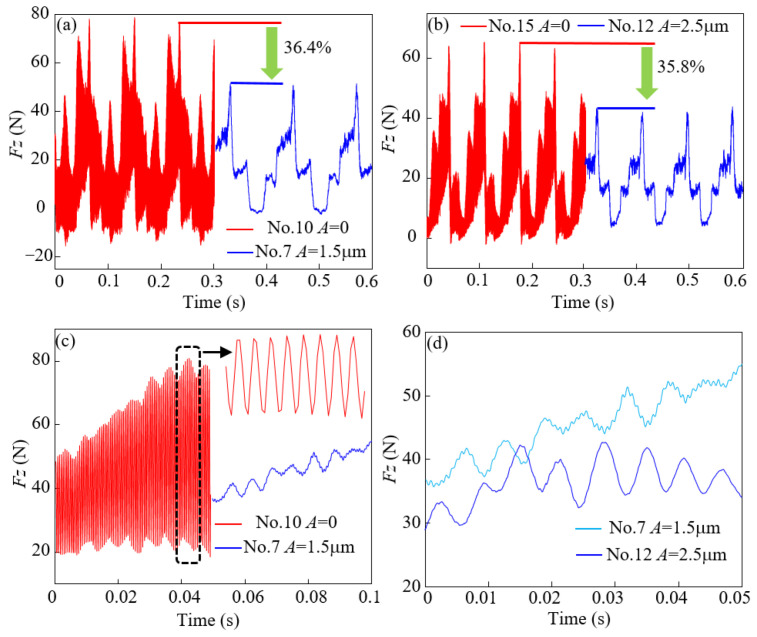
Milling force signal acquisition diagram. (**a**) shows the comparison of milling force for amplitude A = 1.5 μm and A = 0. (**b**) shows the comparison of milling force for amplitude A = 2.5 μm and A = 0. (**c**) compares the sine waveform for amplitude A = 1.5 μm and A = 0. (**d**) compares the sine waveform for amplitude A = 1.5 μm and A = 2.5 μm.

Figure 4 compares the measured cutting forces (*F_z_*) with force sensors under two milling conditions (OM and UAM). Using ultrasonic-assisted milling, the cutting force can be markedly reduced. The results in the 7th group (*v_w_* = 25 m/min, *f_z_* = 20 μm/r, *a_p_* = 0.4 mm, and *A* = 1.5 μm) and the 10th group (*v_w_* = 35 m/min, *f_z_* = 12 μm/r, *a_p_* = 0.4 mm, and *A* = 0 μm) of tests were selected for in-depth analysis, as shown in Figure 4a. Generally, *F_z_* can increase with increasing milling speed and feed per tooth. After the addition of ultrasonic-assisted vibration, the cutter tooth can retain contact and separation characteristics during the rotation process, which can change the total contact time between the cutter tool and the specimen to be processed, thereby playing the role of peak clipping for the cutting force, i.e., the peak of the milling force (*F_z_*) can be reduced. Next, the results in the 7th group (*v_w_* = 35 m/min, *f_z_* = 28 μm/r, *a_p_* = 0.2 mm, and *A* = 2.5 μm) and the 10th group (*v_w_* = 35 m/min, *f_z_* = 12 μm/r, *a_p_* = 0.4 mm, and *A* = 0 μm) of tests were selected for further comparison. Adding the ultrasonic vibration amplitude can still reduce the milling force *F_z_*. It can be observed from Figure 4a,b that ultrasonic-assisted milling can reduce the peak milling force *F_z_* by 36.4% and 35.8%, respectively. Using longitudinal ultrasonic-assisted vibration, the acquired milling force signal can be converted into a pulse signal so that the collected force signal shows the oscillation characteristics featured by a sinusoidal wave, as shown in Figure 4c,d. It can be observed from Figure 4c that before the addition of ultrasonic-assisted milling, no force was applied to the specimen after the signal tooth, and the horizontal rotational inertia was greatest when the cutter tool contacted the TC18 alloy, accompanied by the generation of resonance. Accordingly, the collected milling force shows a large amplitude and sharp peaks. After adding longitudinal ultrasonic vibration, the cutter tool took sinusoidal motion in the vertical direction, which can offset the negative impact induced by horizontal rotational inertia. The peak of the milling force signal became smoother. The sinusoidal profile became more evident with increasing vibration amplitude, as shown in Figure 4d.

Table 3 lists the variance analysis results of the cutting forces along different directions. It can be observed that the feed per tooth imposed significant effects on *F_x_* and *F_y_*, while the cutting depth can significantly affect *F_x_*.

**Figure 5:**
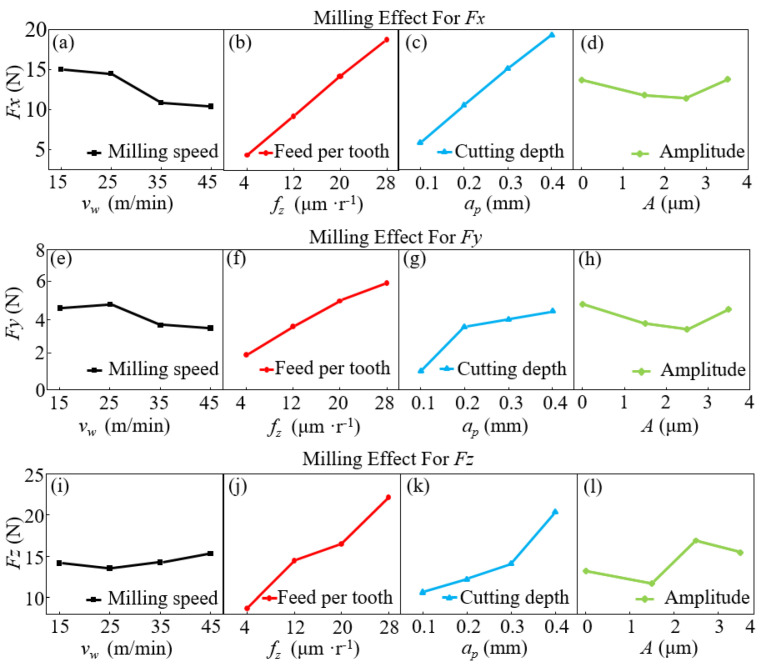
Effects of various milling parameters on the milling force. (**a**–**d**) show the trends of milling speed, feed per tooth, milling depth, and ultrasonic amplitude on milling force Fx, (**e**–**h**) show the trends of milling speed, feed per tooth, milling depth, and ultrasonic amplitude on milling force Fy, and (**i**–**l**) show the trends of milling speed, feed per tooth, milling depth, and ultrasonic amplitude on milling force Fz, respectively.

Figure 5 displays the effects of various cutting parameters on the cutting forces along different directions (*F_x_*, *F_y_,* and *F_z_*). As the milling speed increased, *F_x_* and *F_y_* dropped slightly, and *F_z_* increased slightly, as shown in Figure 5a,e,i. As the feed and cutting depth increased, the cutting forces *F_x_*, *F_y_,* and *F_z_* increased significantly (as shown in Figure 5b,c,f,g,j,k. As the vibration amplitude increased, *F_x_* and *F_y_* first dropped slightly. As the vibration amplitude increased to 3.5 mm, the vibration displacement increased during the feeding process of the cutter tool, and the total contact time between the cutter tool and the specimen was prolonged, increasing *F_x_* and *F_y_*, as shown in Figure 5d,h. Due to the longitudinal ultrasonic vibration of the cutter tool [27], *F_z_* dropped significantly. As the amplitude increased, the displacement between the cutter tool and the specimen increased, accompanied by increased generated longitudinal contact force. Accordingly, *F_z_* increased to a certain degree. However, due to the effect of system stability, a further increase in the amplitude may gradually reduce the cutting force *F_z_*. Through comprehensive evaluation, adding ultrasonic vibration can reduce cutting force by 15% on average.

### 3.2. Milling Temperature

**Figure 6:**
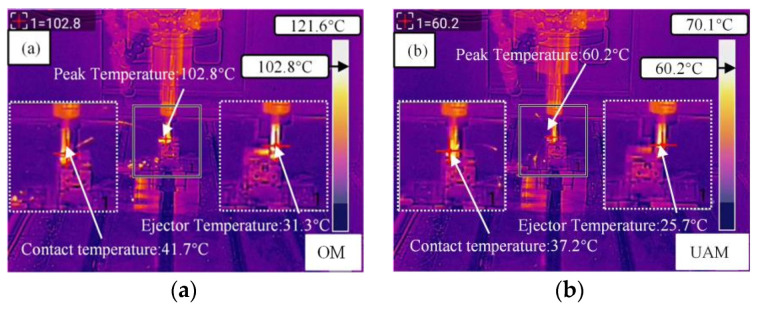
Comparison of the variation in peak milling temperature using OM and UAM. (**a**) shows the temperature variation using OM, including the incoming temperature, the working temperature, and the receding tool temperature. (**b**) shows the temperature variation using UAM, including incoming temperature, working temperature, and retired tool temperature.

Figure 6 compares the milling temperature variations using OM and UAM. As shown in Figure 6a,b, after the addition of ultrasonic vibration, the cutting force was lowered, and the heat dissipation time for the separation of chippings during the vibration of the cutter tool and specimen was prolonged. Accordingly, in contrast with the OM results, the instantaneous friction coefficient dropped, and the milling temperature was significantly reduced, by approximately 42%. With increasing ultrasonic vibration amplitude, the mean temperature increased. At an ultrasonic vibration amplitude of 3.5 μm, the surface temperature of the specimen can be effectively improved [28].

**Figure 7:**
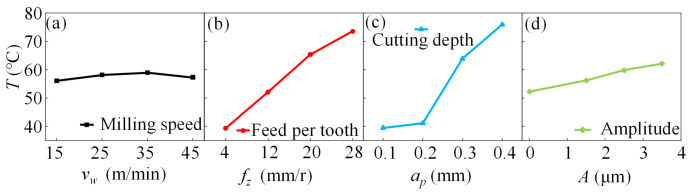
Influencing tendencies of various milling parameters on mean temperature. (**a**) shows the effect of milling speed on temperature, (**b**) shows the effect of feed per tooth on temperature, (**c**) shows the effect of milling depth on temperature, and (**d**) shows the effect of ultrasonic amplitude on temperature.

Figure 7 illustrates the effects of various milling parameters on the mean surface temperature of the specimen. As the feed increased, the contact displacement between the tooth per edge and the specimen increased, and the mean frictional coefficient increased, increasing the mean milling temperature. With increasing milling depth, the cutting force increased, causing a rapid increase in the milling temperature. In contrast, the milling speed slightly affected the mean temperature.

### 3.3. Residual Stress

Residual stress may persist on the specimen surface after the ultrasonic-assisted milling process of TC18. According to the experimental results in Table 2, ultrasonic-assisted milling can enhance this residual stress on the surface. Table 4 and Table 5 list the variance and range analysis results of various technological parameters, from which it can be easily observed that the amplitude of ultrasonic vibration imposed the most significant effect on the residual compressive stress, followed by the milling speed.

**Figure 8:**
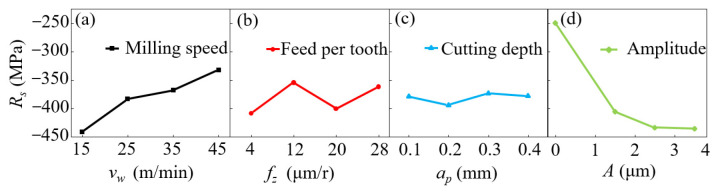
Influencing tendencies of various milling parameters on residual stress. (**a**) shows the effect of milling speed on residual stresses, (**b**) shows the effect of feed per tooth on residual stresses, (**c**) shows the effect of milling depth on residual stresses, and (**d**) shows the effect of ultrasonic amplitude on residual stresses.

Figure 8 displays the effects of various milling parameters on the residual compressive stress using ultrasonic-assisted milling. Since TC18 is a hard, difficult-to-process alloy, the residual compressive stress was high at low cutting speeds; as the cutting speed increased, the residual compressive stress dropped. With increasing ultrasonic vibration amplitude, the residual compressive stress on the surface of the Ti alloy specimen increased rapidly, with a mean extreme of up to 430 MPa. This can be attributed to the impact of ultrasonic vibration on the specimen during the milling process. Accordingly, after adding ultrasonic vibration, the residual stress on the specimen was markedly enhanced compared with the value using ordinary milling.

### 3.4. Surface Roughness

**Figure 9:**
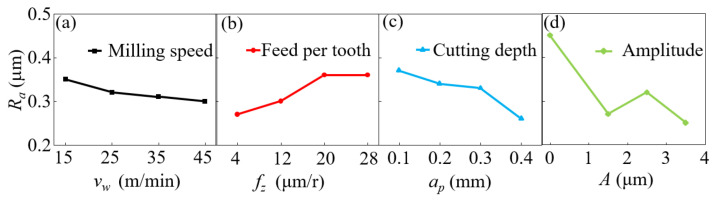
Influencing tendencies of various milling parameters on surface roughness. (**a**) shows the effect of milling speed on roughness, (**b**) shows the effect of feed per tooth on roughness, (**c**) shows the effect of milling depth on roughness, and (**d**) shows the effect of ultrasonic amplitude on roughness.

Figure 9 shows the variations in the surface roughness with the cutting speed *v_w_*, the feed *f_z_*, the cutting depth *a_p_* and the amplitude of ultrasonic vibration *A*. As the cutting speed and the cutting depth increased, the surface roughness of the specimen dropped; as the feed increased, the mean roughness first increased by up to 30%; as the vibration of ultrasonic vibration increased, the surface roughness improved and reached an optimal value at an amplitude of 3.5 μm. Under optimal ultrasonic-assisted milling conditions, the surface roughness can be reduced by 44% compared with the value under ordinary milling conditions.

### 3.5. Surface Morphology

**Figure 10:**
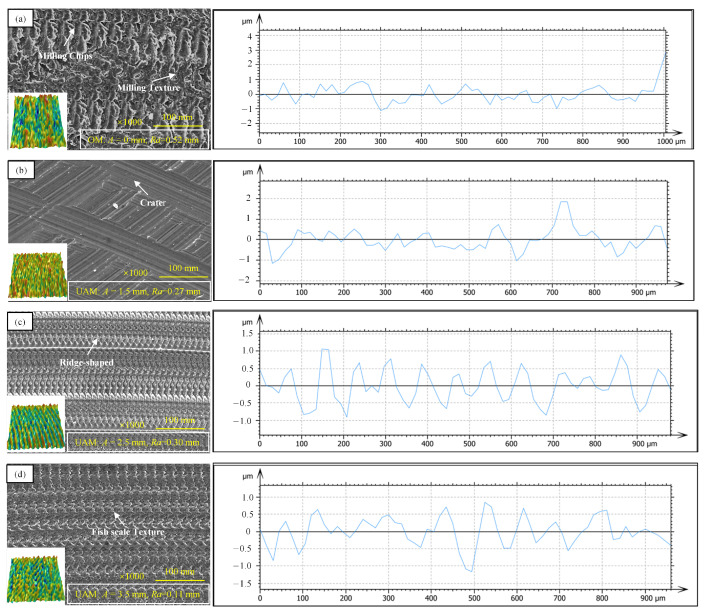
Surface topographical patterns and roughness degrees of the specimens after different milling processes. (**a**) shows the surface profile and surface roughness of conventional milling, (**b**) shows the surface profile and surface roughness of amplitude A = 1.5 μm, (**c**) shows the surface profile and surface roughness of amplitude A = 2.5 μm, and (**d**) shows the surface profile and surface roughness of amplitude A = 3.5 μm.

Using a COXEM desk-type scanning electron microscope and white light interferometer, the surface morphological patterns of the specimens were observed at a magnification of 1000, as shown in Figure 10.

Figure 10a shows the surface morphology of the TC18 specimen using ordinary milling (at zero amplitude of ultrasonic vibration). Banded wavy textures can be observed along the feeding direction of the cutter tool. Figure 10b shows the surface morphology of the TC18 specimen after ultrasonic-assisted milling at an amplitude of 1.5 μm. The surface roughness can be reduced, and the microtextures on the surface show a regular distribution along the feeding direction. Groove structures can be observed at the cutting position of the cutter tool. The existence of microtexture can contribute to the storage of lubricating medium so that lubricating medium can create an interception effect between friction pairs, thereby improving the friction performance of friction pairs. Figure 10c shows the surface morphology of the TC18 specimen after ultrasonic-assisted milling at an amplitude of 2.5 μm, from which a ridged sinusoidal distribution of tight textures along the feeding direction can be observed. As the vibration amplitude increased, the total roughness increased, but the peak roughness dropped. The existence of ridged textures in the friction pairs of Ti alloy bearings can generate adhesive friction, forming anisotropy of the friction force. As the amplitude of ultrasonic vibration increased to 3.5 μm, the surface morphology of the TC18 specimen after ultrasonic-assisted milling was observed, as shown in Figure 10d. Regularly distributed fish scale textures can be observed. Slight roughness and regular surface textures can improve both the friction force and consumption to a certain degree.

### 3.6. Optimized Combination of Technological Parameters

During the ultrasonic-assisted milling process of the TC18 alloy, the combinations of milling parameters were concluded to achieve good processing performance, as listed in Table 6. Aiming at obtaining intact surface textures and low surface roughness, the A_4_B_1_C_4_D_4_ combination of technological parameters (at a milling speed of 45 m/min, a feed of 4 μm/r, a cutting depth of 0.4 mm and an ultrasonic vibration amplitude of 0.35 μm) can be selected. For the enhancement of residual compressive stress on the surface, the A_1_B_1_C_2_D_4_ combination can be selected. To reduce the milling temperature, the A_1_B_1_C_1_D_4_ combination can be selected.

## 4. Conclusions

The results obtained made it possible to draw the following conclusions:

(1) By comparing the machining effects under ordinary milling and longitudinal ultrasonic-assisted milling, it can be found that the mean cutting force can be reduced by 15% due to high-speed contact and separation between the longitudinal ultrasonic vibration cutter tool and the workpiece, thereby effectively enhancing the cutting performance.
(2) During the longitudinal ultrasonic vibration milling process, prolonging the separation time between the cutter tool and the workpiece can increase the heat dissipation time, remarkably improving the temperature rise during the milling process. The peak temperature can be reduced by up to 42%.
(3) Adding longitudinal ultrasonic vibration to the milling process can enhance the residual compressive stress on the specimen surface. The improvement became more evident with increasing amplitude. The peak residual compressive stress can be enhanced by approximately 40%.
(4) Ultrasonic-assisted milling can remarkably improve the surface roughness of TC18 alloys. The maximum reduction of the surface roughness can reach 44% at an ultrasonic vibration amplitude of 3.5 μm.
(5) Prospects for future work: after orthogonal test analysis, some processing parameters on TC18 were accumulated. The subsequent experiments can be expanded based on the excellent processing parameters. Single-factor tests were conducted to investigate the in-depth effects of each parameter on TC18 milling, the effect of machining parameters on force, and thus the difference in surface integrity. Enrichment of the orthogonal test data to obtain the interactive effects of each parameter on force and surface integrity should be considered.

## Figures and Tables

**Table 1:** Chemical composition of TC18 alloy (mass fraction).

Project	Chemical Composition											
Composition	Ti	Al	Mo	V	Cr	Fe	Si	C	N	O	H	Zr
Mass fraction/%	Residuals	5.32	5.14	5.07	1.03	1.05	0.024	0.014	0.004	0.1	0.002	<0.01

**Table 2:** Orthogonal test results.

No.	Factor A *v_w_* (m/min)	Factor B *f_z_* (μm/r)	Factor C *a_p_* (mm)	Factor D *A* (μm)	*F_x_*/N	*F_y_*/N	*F_z_*/N	*R_s_*/MPa	*T*/°C	*R_a_*/μm
1	15	4	0.1	0	2.48	−0.53	4.18	−318.61	22.4	0.49
2	15	12	0.2	1.5	7.93	−1.93	8.36	−450.29	40.3	0.32
3	15	20	0.3	2.5	17.68	−5.56	15.00	−502.25	67.3	0.30
4	15	28	0.4	3.5	31.92	−10.60	29.13	−492.44	94.2	0.30
5	25	4	0.2	3.5	4.24	−1.75	6.77	−495.84	34.9	0.11
6	25	12	0.1	2.5	4.66	−0.70	7.27	−406.55	34	0.40
7	25	20	0.4	1.5	23.64	−9.18	19.60	−267.26	76.6	0.27
8	25	28	0.3	0	25.38	−7.86	20.55	−363.96	87	0.51
9	35	4	0.3	1.5	4.98	−2.36	7.68	−440.48	49.2	0.25
10	35	12	0.4	0	14.82	−6.97	24.10	−374.08	82	0.24
11	35	20	0.1	3.5	7.34	−1.29	9.39	−429.84	46.5	0.34
12	35	28	0.2	2.5	16.40	−4.19	15.92	−227.66	58	0.38
13	45	4	0.4	2.5	6.80	−3.34	8.62	−378.90	51	0.21
14	45	12	0.3	3.5	12.36	−4.83	13.25	−186.25	52	0.23
15	45	20	0.2	0	13.66	−4.22	17.86	−401.75	71.2	0.52
16	45	28	0.1	1.5	8.85	−1.70	21.70	−362.05	55	0.25

**Table 3:** Significance analysis of the milling force.

Factor	Degree	Sum of Squares of Deviations			*F*-Ratio			Significance		
		*F_x_*	*F_y_*	*F_z_*	*F_x_*	*F_y_*	*F_z_*	*F_x_*	*F_y_*	*F_z_*
*v_w_* (m/min)	3	67.78	5.46	6.76	2.39	0.98	0.14	0	0	0
*f_z_* (mm/r)	3	576.52	38.18	460.73	20.33	6.88	9.23	Significant	Significant	0
*a_p_* (mm)	3	404.08	92.84	218.08	14.25	16.74	4.37	Significant	0	0
A (μm)	3	18.65	2.80	63.47	0.66	0.50	1.27	0	0	0
Error	3	28.36	5.55	49.92						

**Table 4:** Significance analysis results of the residual stress.

Factor	Sum of Squares of Deviations	Degree	F-Ratio	Significance
*v_w_* (m/min)	24,559.19	3	9.05	0
*f_z_* (mm/r)	8871.33	3	3.27	0
*a_p_*/mm	949.08	3	0.35	0
*A*/μm	93,995.35	3	34.65	Significant
Error	2712.87	3		

**Table 5:** Range analysis results of the residual stress.

Average Value	*v_w_* (m/min)	*f_z_* (μm/r)	*a_p_*/mm	*A*/μm
K_1_	−1763.6	−1633.8	−1517.1	−999.8
K_2_	−1533.6	−1417.2	−1575.5	−1623.0
K_3_	−1472.1	−1601.1	−1492.9	−1734.2
K_4_	−1328.9	−1446.1	−1512.7	−1741.2
Range	108.7	54.16	20.65	185.4
Decreasing order of factors		*A* > *v_w_* > *f_z_* > *a_p_*		
Optimal combination		A_1_B_1_C_2_D_4_		

**Table 6:** Optimization combinations of technological parameters.

Optimized Parameter	Optimal Combination
Cutting force *F_x_*	A_4_B_1_C_1_D_3_
Cutting force *F_y_*	A_4_B_1_C_1_D_3_
Cutting force *F_z_*	A_2_B_1_C_1_D_2_
Milling temperature	A_1_B_1_C_1_D_4_
Residual stress	A_1_B_1_C_2_D_4_
Surface roughness	A_4_B_1_C_4_D_4_

## Data Availability

All relevant data can be obtained in this article.
